# Dairy cows inoculated with highly pathogenic avian influenza virus H5N1

**DOI:** 10.1038/s41586-024-08166-6

**Published:** 2024-10-15

**Authors:** Amy L. Baker, Bailey Arruda, Mitchell V. Palmer, Paola Boggiatto, Kaitlyn Sarlo Davila, Alexandra Buckley, Giovana Ciacci Zanella, Celeste A. Snyder, Tavis K. Anderson, Carl R. Hutter, Thao-Quyen Nguyen, Alexey Markin, Kristina Lantz, Erin A. Posey, Mia Kim Torchetti, Suelee Robbe-Austerman, Drew R. Magstadt, Patrick J. Gorden

**Affiliations:** 1 Virus and Prion Research Unit, National Animal Disease Center, Agricultural Research Service, United States Department of Agriculture, Ames, IA USA; 2 National Veterinary Services Laboratories, Animal and Plant Health Inspection Service, United States Department of Agriculture, Ames, IA USA; 3 Veterinary Diagnostic and Production Animal Medicine, Iowa State University, Ames, IA USA

**Keywords:** Influenza virus, Viral pathogenesis

## Abstract

Highly pathogenic avian influenza (HPAI) H5N1 haemagglutinin clade 2.3.4.4b was detected in the USA in 2021. These HPAI viruses caused mortality events in poultry, wild birds and wild mammals. On 25 March 2024, HPAI H5N1 clade 2.3.4.4b was confirmed in a dairy cow in Texas in response to a multistate investigation into milk production losses^1^. More than 200 positive herds were identified in 14 US states. The case description included reduced feed intake and rumen motility in lactating cows, decreased milk production and thick yellow milk^2,3^. The diagnostic investigation revealed viral RNA in milk and alveolar epithelial degeneration and necrosis and positive immunoreactivity of glandular epithelium in mammary tissue. A single transmission event, probably from birds, was followed by limited local transmission and onward horizontal transmission of H5N1 clade 2.3.4.4b genotype B3.13 (ref. ^4^). Here we sought to experimentally reproduce infection with genotype B3.13 in Holstein yearling heifers and lactating cows. Heifers were inoculated by an aerosol respiratory route and cows by an intramammary route. Clinical disease was mild in heifers, but infection was confirmed by virus detection, lesions and seroconversion. Clinical disease in lactating cows included decreased rumen motility, changes to milk appearance and production losses. Infection was confirmed by high levels of viral RNA detected in milk, virus isolation, lesions in mammary tissue and seroconversion. This study provides the foundation to investigate additional routes of infection, pathogenesis, transmission and intervention strategies.

## Abstract (web summary)

A study describes the experimental infection of cattle with a highly pathogenic avian influenza H5N1 clade 2.3.4.4b genotype B3.13 strain using an aerosol respiratory route for heifers and an intramammary route for lactating cows.

## Main

HPAI H5N1 viruses of the goose/Guangdong lineage in the haemagglutinin clade 2.3.4.4b were detected in the USA in 2021 following widespread dispersion across Asia and Europe^5^. H5N1 viruses in this clade were detected across the USA in mortality events in wild bird species and wild mammals. Additionally, outbreaks occurred in numerous commercial and backyard poultry premises, leading to culling to control the spread. On 25 March 2024, the National Veterinary Services Laboratories of the US Department of Agriculture confirmed a case of HPAI H5N1 clade 2.3.4.4b genotype B3.13 in a dairy cow in Texas in response to a multistate investigation into milk production losses. Additional outbreaks were rapidly identified in Texas as well as eight other US states. As of 12 September 2024, more than 200 cases had been confirmed in 14 states^1^. The typical case description on the affected dairy farms included reduced feed intake and rumination in lactating cows, rapid drop in milk production, and affected cows with thick yellow milk with flecks and/or clots^2,3^. The diagnostic investigation revealed low cycle threshold (Ct; high viral load) detections of viral RNA by quantitative real-time PCR with reverse transcription (RT–qPCR) in milk, mammary tissue with alveolar epithelial degeneration and necrosis with intraluminal sloughing of cellular debris, and positive immunoreactivity of glandular epithelium with nuclear and cytoplasmic labelling by immunohistochemistry (IHC). Deceased domestic cats located on affected dairy farms that presented with neurologic and respiratory signs were also confirmed to be infected with the same H5N1 clade 2.3.4.4b B3.13 genotype, as were several peridomestic wild birds found around or near affected premises.

A genomic and epidemiologic investigation demonstrated that a single transmission event from avian species preceded the multistate cattle outbreak^4^. The movement of preclinical or subclinical lactating dairy cows was the main contributor to spread among the US dairy premises. Sequence analysis thus far revealed highly similar genomes among cattle detections with little evolution or known mammalian adaptation markers^4^. Several human infections were reported with H5N1 clade 2.3.4.4b, and although some were severe or fatal, the human detections thus far associated with the dairy outbreak in the USA were mild^6–8^. However, transmission in multiple host species, particularly mammals, raises the concern for mammalian adaptation that may lead to increased potential for human infection and/or transmission.

Although a few sporadic detections of human seasonal influenza A virus (IAV) or antibodies were previously reported in experimental inoculation of dairy cows^9,10^ or associated with milk production loss^11,12^, the Texas cases in 2024 were the first reports of HPAI of any subtype causing viral mastitis in lactating dairy cows. At the time we initiated these experiments, the route of infection and transmission between cows was unknown. Transmission between farms was linked to movement of live lactating cows, yet within-farm spread to resident cows was observed within days or weeks following movement without a clear pattern of transmission consistent on all farms. Here we sought to experimentally reproduce infection of dairy cattle with genotype B3.13 in Holstein yearling heifers through an aerosol respiratory route and in lactating cows through an intramammary route.

## Phylogenetic analysis of study strain

**Extended Data Fig. 1:**
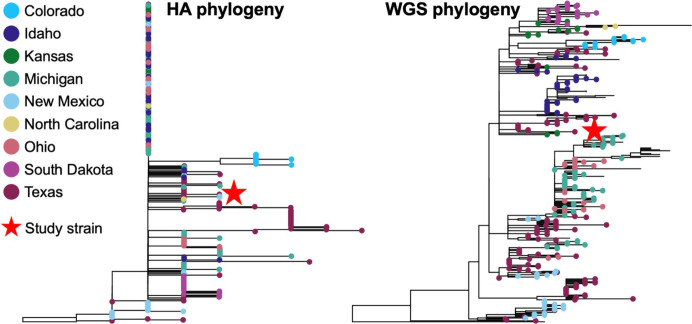
Evolutionary history of H5N1 clade 2.3.4.4b hemagglutinin genes from dairy cattle in the United States between March and May 2024. The HA gene and whole genome phylogeny demonstrated a single introduction of the virus from wild birds to dairy cattle with subsequent dissemination across nine US states. The A/dairy cattle/Texas/24-008749-002/2024 study strain, indicated by a red star, was 99.94% identical to a B3.13 whole genome consensus strain (9 nucleotide substitutions across the genome); and at the amino acid level, the TX/24 HA protein was identical to a consensus B3.13 HA sequence, with only 2 synonymous substitutions detected at the nucleotide level.

The phylogenetic tree topology (Extended Data Fig. 1) with B3.13 genotype strains collected from dairy cattle between March and May 2024 was congruent with earlier analyses^4^. The HA gene tree indicated a single monophyletic clade; following the introduction of the B3.13 virus genotype into dairy cattle in late 2023 (ref. ^4^), it persisted and rapidly spread through cattle populations. There was no evidence of reassortment, and all viruses detected in dairy cattle were classified as B3.13 genotype maintaining the new polymerase basic 2 (PB2) and nucleoprotein (NP) gene that had been acquired in migratory birds before the spillover^4^. The HA gene tree and the phylogenomic tree had little evidence for directional selection with long external branches relative to internal branches, and many viruses that were identical. These data suggested a founder effect in which a single genotype was introduced into a new population of susceptible hosts^13^. The TX/24 challenge strain was 99.94% identical to a B3.13 whole-genome consensus strain (nine nucleotide substitutions across the genome); the TX/24 HA gene had only two synonymous substitutions and the protein was identical to a consensus B3.13 HA amino acid sequence.

## Clinical observations

**Fig. 1:**
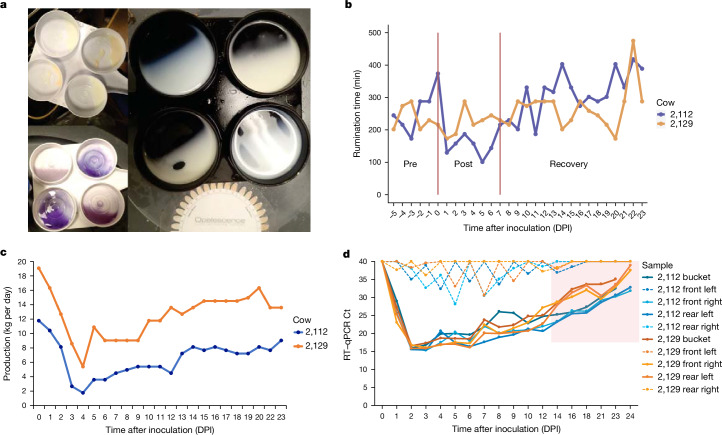
Clinical signs and viral detection in lactating dairy cows. **a**, Representative milk sampling demonstrating thickening, flakes or clots, and change in colour in the upper left and right photographs, and gel formation in positive California mastitis tests in the lower left photograph. In all photos, changes were observed in the inoculated front right and rear left quarters in the upper right and lower left cups of the milk collection paddle in each photo. **b**, Daily rumination time in minutes for cow 2,112 (blue) and cow 2,129 (orange) measured using an ear-tag accelerometer sensor. Rumination time decreased at 1 DPI and recovered to pre-challenged levels at 7 DPI. **c**, Each individual milking machine bucket was weighed once daily to monitor production, for cow 2,112 (blue) and cow 2,129 (orange). Milk production steadily declined from 1 to 4 DPI, remained low until 10–12 DPI, and was 71–77% of pre-inoculation production at 23 DPI. **d**, Milk was stripped from each teat before milking and a sample was taken from the bucket after milking, for cow 2,112 (blue) and cow 2,129 (orange). An RT–qPCR assay detected viral RNA beginning on 1 DPI until study termination at 24 DPI in the inoculated quarters (solid lines), with Ct on the *y* axis and DPI on the *x* axis. The positive detections in non-inoculated quarters (dashed lines) were probably cross-contamination due to the non-sterile collection of milk from each teat. The pink shading indicates time points when live virus isolation was attempted from the bucket samples and samples were negative by egg inoculation. One inoculated quarter from cow 2,112 was positive for virus isolation on 12 DPI, but all tested samples beyond 12 DPI were negative for virus isolation.

Following transition to containment, rumen motility, feed consumption and milk production stabilized in the lactating cows by the time of inoculation. Following inoculation, both cows showed signs of mastitis with a positive California mastitis test and milk colour and consistency changes beginning at 2 days post inoculation (DPI) and lasting until approximately 14 DPI, only in the inoculated quarters (Fig. 1a). The peak average colour change score in the milk from inoculated quarters from both cows was 6.5 on a yellow to brown scale (0–12) on 5 and 6 DPI. The milk was yellow, thicker and contained flakes and clots of debris from affected quarters during the same time period. Rumen motility markedly declined on 1 DPI and began recovering after 7 DPI (Fig. 1b). Milk production steadily declined from 1 to 4 DPI, remained low until 10–12 DPI, and was 71–77% of pre-inoculation production (0 DPI) at 23 DPI (Fig. 1c). The cows showed signs of lethargy, reduced feed intake, self-resolving watery diarrhoea or dry faeces, and intermittent clear nasal discharge throughout the 24-day observation period. Neurologic signs were not observed.

The heifer calves exhibited no overt signs of illness, with only transient increases in nasal secretions observed. Other clinical parameters indicative of illness were not consistently observed in the lactating cows or heifer calves.

## Virus detection

Milk stripped from inoculated mammary quarters and the post-milking bucket sample were continuously positive by RT–qPCR from 1 DPI until the end of the study (Fig. 1d). Although stripped milk from non-inoculated quarters had a Ct below 35, the detection was not consistent over consecutive days and never progressed to low Ct values indicative of live virus replication in those mammary glands. Milk bucket samples subjected to virus isolation were positive on 5, 7 and 10 DPI and negative on 12, 14, 16 and 18 DPI. One inoculated quarter from cow 2,112 was positive for virus isolation on 12 DPI, but all tested mammary quarter samples beyond 12 DPI were negative for virus isolation.

**Fig. 2:**
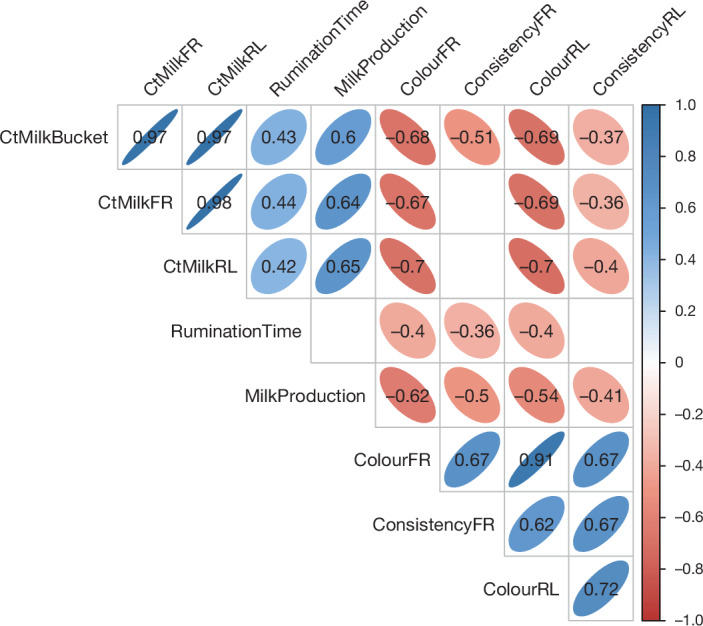
Correlation between viral RNA detection and clinical signs. Significant (*P* < 0.05, two-sided) Pearson correlation coefficients between Ct values for milk samples from the bucket (*n* = 34) as well as the inoculated teats (front right (FR) (*n* = 34) and rear left (RL) (*n* = 34)) and rumination time (*n* = 46), milk production (*n* = 44) and consistency (*n* = 48) and colour (*n* = 48) scores of milk from the inoculated teats across all time points. Empty squares in the matrix indicate the correlation was not significant (*P* < 0.05, two-sided).

There were strong positive correlation coefficients between Ct values from the milk bucket and infected quarters with rumination and milk production. As Ct decreased (higher viral load) so did rumination and milk production. There were also strong negative correlation coefficients between milk Ct values and colour and consistency scoring, as Ct decreased (higher viral load), colour and consistency scores increased (Fig. 2). Only one nasal swab (Ct 33.1) from cow 2,112 and one ocular swab (Ct 34.7) from cow 2,129 on 3 DPI had RT–qPCR Ct ≤ 35; all remaining clinical samples had RT–qPCR Ct greater than 35 cycles or undetected. No faecal swab or blood samples were positive by RT–qPCR at any time point. From the broad range of samples collected at necropsy, mammary tissue from inoculated quarters and supramammary lymph nodes had RT–qPCR Ct ≤ 35, and an inguinal lymph node and one inoculated mammary tissue had a Ct of between 35 and 38. All other samples were not within RT–qPCR detection limits. (Extended Data Table 1).

Nasal, oropharyngeal, saliva and ocular samples collected from the aerosol-inoculated heifers sporadically had RT–qPCR Ct ≤ 35 (Extended Data Table 2). Heifer 2,316 had nasal swabs with Ct ≤ 35 from 1 to 5 DPI and at 7 DPI. Viral RNA was also detected across multiple days in oropharyngeal swabs, ocular swabs or saliva from heifer 2,316. Viable virus isolation was attempted on clinical samples with Ct ≤ 35, and 2–5- and 7-DPI nasal swabs from heifer 2,316 were virus isolation positive. This heifer was euthanized for necropsy on 7 DPI, so no further ante-mortem samples were collected. Each of the other three heifers tested positive by RT–qPCR on at least one time point between 1 and 7 DPI, but sporadically and not on consecutive days. Heifer 2,311 had one FLOQ oropharyngeal swab that was positive for virus isolation on 3 DPI. No faecal swab or blood samples were positive by RT–qPCR at any time point. The sham-inoculated negative control heifer 2,309 had no RT–qPCR detections as expected. From the broad range of samples collected at the 7-DPI necropsy, lung tissue from both heifers (accessory lobes and cranial part of the left cranial lobe) had RT–qPCR Ct ≤ 35, and a retropharyngeal lymph node, a lung lobe (caudal part of the right cranial lobe and middle lobe) and a turbinate sample had a Ct between 35 and 38. All other samples at DPI 7 as well as all samples from the 20-DPI necropsy were not within RT–qPCR detection limits (Extended Data Table 2).

## Single nucleotide variant analysis of samples

Across 82 clinical samples collected from the experimentally inoculated cattle, we identified within-host single nucleotide variants that were present at low frequencies (present in greater than 0.5%) across the genome. We matched a custom database of variants of interest that could potentially provide a selective advantage and alter virus phenotype. Overall, there were 3,960 single nucleotide variants present at low frequencies, of which 2,676 altered the amino acid with nonsynonymous changes (Extended Data Table 3). Of the amino-acid changes detected, only 12 had previously been associated with known functional changes. We detected low-frequency variants associated with changes in pathogenicity or virulence in the gene segments encoding matrix (T139A), non-structural (T91N, T91A, D92E, D92N, T94A, L95P, S99P, D101N, D101G and D125N) and PB1 (R622Q) proteins. We also detected low-frequency variants associated with mammal adaptation in non-structural (F103S) and PB2 (L631P) proteins. Despite these low-frequency detections, none had allele frequencies greater than 11%; however, if any such mutations provided a selective advantage, this phenotype could be selected for increased frequency in the population. There was a marginal trend for more variants and increased nonsynonymous sites on DPI 1–3 and 9–14; however, this was not significant as the sample sizes were small, and the differences had overlapping ranges (Extended Data Table 3).

## Antibody responses

The dairy cows inoculated by the intramammary route were negative for NP antibody in serum before inoculation, became positive on 7 DPI using a sample/negative ratio cutoff of 0.6, and remained positive on all subsequent sample time points (Extended Data Table 4a). Fresh stripped milk from both cows was also positive by NP enzyme-linked immunosorbent assay (ELISA) by 9 DPI. Both cows were seropositive by haemagglutinin inhibition (HI) assay on 24 DPI and by virus neutralization antibody assay on 14 and 24 DPI (Extended Data Table 4b). Milk samples collected from each quarter on 24 DPI were tested by HI with reciprocal titres of 10–40, with both cows having quarters above and below the HI positive cutoff of ≥40. However, virus neutralization antibody assays in inoculated quarters were positive on 9 DPI for cow 2,129 and on 12 DPI for cow 2,112. The uninoculated quarters from both cows remained below the positive cutoff titre of 1:40. Two of the heifer calves were positive for NP antibodies on 7 DPI, and the remaining two seroconverted between 9 and 13 DPI (Extended Data Table 4c). One calf was positive by HI assay and the calf with a later NP antibody response was suspect by HI assay at 20 DPI. The sham-inoculated negative control remained seronegative as expected.

## Pathologic evaluation

**Extended Data Fig. 2:**
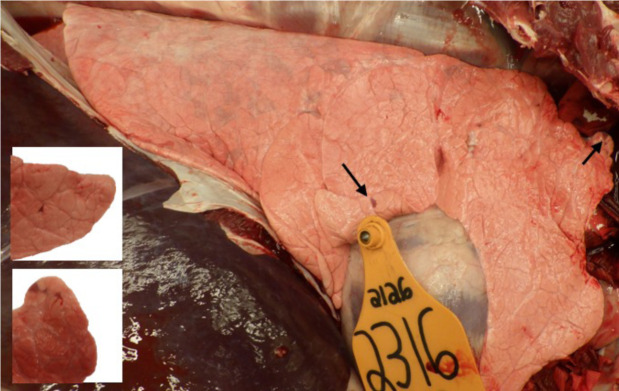
Macroscopic lung lesions in a heifer. Minimal multifocal obstructive atelectasis (arrows and insets) consistent with influenza A virus infection in Heifer 2316.

Minimal multifocal obstructive atelectasis was present in heifer 2,316 (Extended Data Fig. 2). In the lactating cows, lesions considered incidental were observed and included a unilateral locally extensive pleural adhesion and transparent, straw-coloured abdominal fluid in cow 2,112 and interlobular pulmonary oedema in cow 2,129. Macroscopic evaluation of the remaining tissues was unremarkable.

**Fig. 3:**
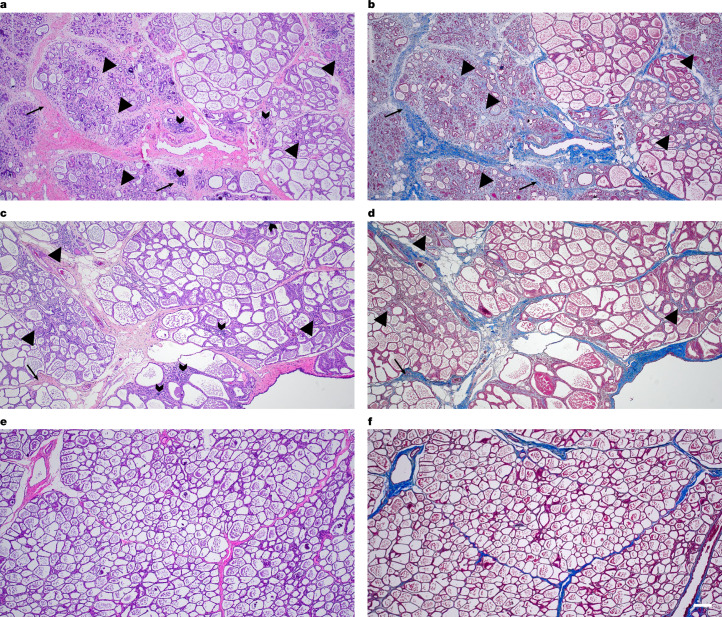
Fibrosis in inoculated mammary gland tissue. **a**,**c**, Representative photomicrographs of the interlobular (arrowheads) and intralobular (arrows) fibrosis and inflammatory infiltrate (chevrons) of the left rear mammary gland in cows 2,112 (**a**) and 2,129 (**c**). **b**,**d**, A matched Masson’s trichrome stain (fibrosis is blue) demonstrates the extent of fibrosis in cows 2,112 (**b**) and 2,129 (**d**). **e**,**f**, A representative photomicrograph showing haematoxylin–eosin (**e**) and Masson’s trichrome (**f**) staining in a non-inoculated teat (left front) of cow 2,112 for comparison. All photomicrographs are at ×100 magnification and the scale bar (200 μm) applies to all panels.

Similar histologic lesions were present in the left rear mammary gland (inoculated) of both dairy cattle. Fibrous connective tissue replaced 15% to 50% of secretory alveoli and ducts, both intralobular and interlobular, of multifocal lobules (Fig. 3a–d). The remaining secretory alveoli in affected lobules were commonly shrunken and lined by small, attenuated or swollen, vacuolated epithelial cells (atrophy and degeneration). Both interlobular and intralobular fibrous connective tissue contained multifocal aggregates of segmental to circumferential perialveolar or periductular mononuclear inflammatory cells predominated by lymphocytes and plasma cells. The remaining secretory alveoli and ducts contained secretory product admixed with occasional foamy cells (macrophages or foam cells) or rarely deeply basophilic, concentrically lamellated foci (corpora amylacea). Non-affected to minimally affected glandular tissue commonly contained moderately to well-developed secretory alveoli. Histologic evaluation of the non-inoculated mammary quarters (left front and right rear) was characterized by well-developed secretory epithelium that contained fluid and no to minimal intralobular or interlobular fibrosis and no to rare aggregates of mononuclear leukocytes predominated by lymphocytes and plasma cells (Fig. 3e,f). A summary of microscopic findings including IHC is presented in the data file in Zenodo (see Data availability section).

**Fig. 4:**
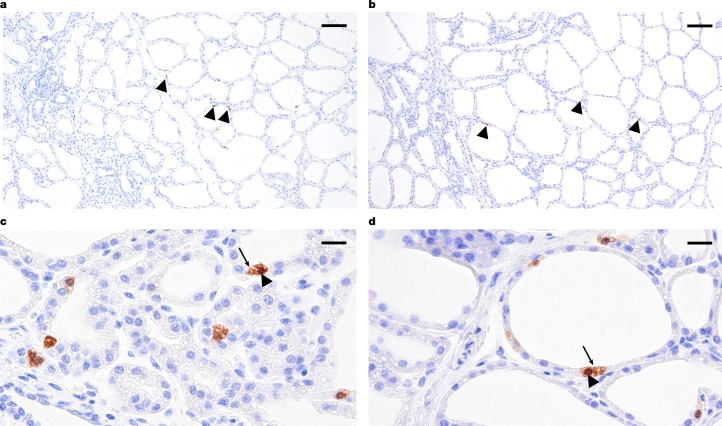
IHC in mammary tissue. **a**–**d**, IHC detection of IAV antigen in epithelial cells lining secretory alveoli of the left rear mammary glands (arrowheads in **a**,**b**) with higher-magnification images showing staining in the cytoplasm (arrows in **c**,**d**) and nucleus (arrowheads in **c**,**d**), for cows 2,112 (**a**,**c**) and 2,129 (**b**,**d**). Photomicrographs at ×100 (**a**,**b**) and ×400 (**c**,**d**) magnification. Scale bars, 100 μm (**a**,**b**) and 20 μm (**c**,**d**).

**Extended Data Fig. 3:**
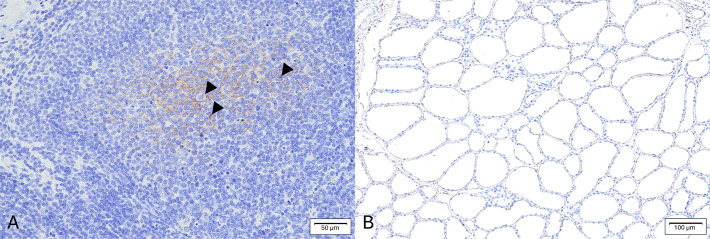
Influenza A virus immunohistochemistry in lymph node and mammary gland. Influenza A virus detection by immunohistochemistry (arrowheads) in the germinal center light zone of the supramammary lymph node of cow 2129 (A). Immunohistochemistry did not detect Influenza A virus antigen in the uninoculated left front mammary gland of 2112 (B). Photomicrographs at 200X (A) and 100X (B) magnification.

IAV NP antigen was detected in the cytoplasm and nucleus of epithelial cells lining secretory alveoli in the inoculated mammary gland quarters of both cows at 24 DPI (Fig. 4a–d). IAV NP antigen was also detected in the light zone of multiple germinal centres of the supramammary lymph node of cow 2,129 (Extended Data Fig. 3).

**Fig. 5:**
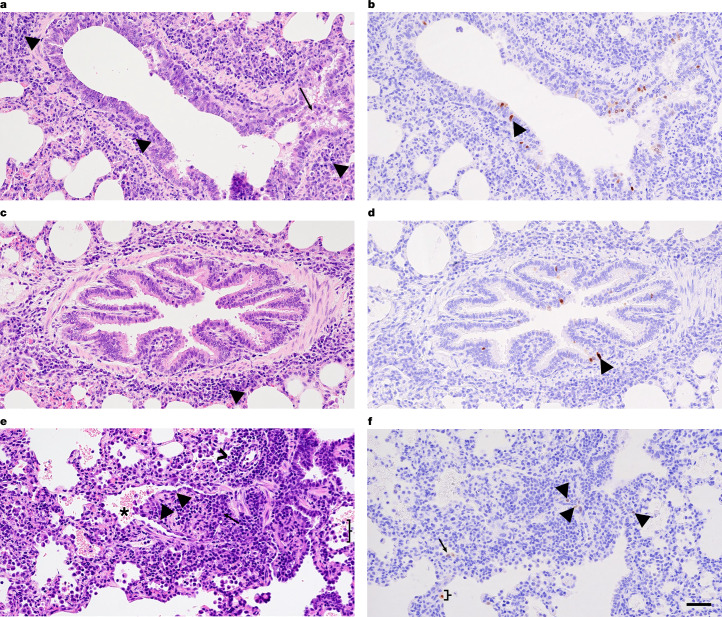
Histopathologic changes in heifer lung tissue. **a**, Peribronchiolar mononuclear cell inflammatory infiltrate (arrowheads) and accumulation of intraluminal inflammatory cells (arrow) in a bronchiole in the cranial part of the left cranial lung lobe of heifer 2,311. **b**, Replication of HPAI virus in the respiratory epithelium lining the affected bronchiole of heifer 2,311 shown in **a**, as demonstrated by IHC (arrowheads). **c**, Peribronchiolar mononuclear cell inflammatory infiltrate (arrowheads) in another bronchiole in the cranial part of the left cranial lung lobe of heifer 2,311. **d**, Replication of HPAI virus in the respiratory epithelium lining the affected bronchiole of heifer 2,311 shown in **c**, as demonstrated by IHC (arrowheads). **e**, Bronchiolitis obliterans in heifer 2,316. The lumen of a bronchiole (asterisk) is partially occluded by a polyp lined by attenuated epithelial cells (arrowheads). The polyp is composed of fibroblasts, fibrin and lymphocytes (arrow). Adjacent alveoli contain increased inflammatory cells (probable alveolar macrophages; brace). An adjacent arteriole is circumferentially surrounded by a mononuclear cell inflammatory infiltrate composed predominately of lymphocytes and fewer plasma cells (chevron). **f**, Replication of HPAI virus in the respiratory epithelium lining the affected bronchiole of heifer 2,316 as demonstrated by IHC (arrowheads), and in probable type II pneumocytes (arrow) and alveolar macrophage (brace). All photomicrographs are at ×200 magnification and the scale bar (50 μm) applies to all panels.

In the heifers necropsied at 7 DPI, minimal lesions consistent with IAV infection were focused on conducting airways. The interstitium of a bronchiole of the caudal part of the left cranial lung lobe of heifer 2,311 was minimally circumferentially expanded by a predominance of lymphocytes and plasma cells. IAV was detected by IHC within the respiratory epithelial cells lining this conducting airway (Fig. 5a–d). Bronchiolitis obliterans was present in the caudal part of the left cranial lung lobe of heifer 2,316 (Fig. 5e). The lumen of a focal bronchiole was 80% occluded by a polyp of fibrocytes and fibrin admixed with inflammatory cells and lined by markedly attenuated epithelium. The surrounding interstitium was mildly circumferentially expanded by lymphocytes and plasma cells. Scant IAV NP antigen was detected in the remaining epithelial cells of the affected bronchiole, adjacent type II pneumocytes and probable alveolar macrophage (Fig. 5f). Additional histologic lesions were observed in both cows and heifers; however, the role of IAV infection in the development of these lesions could not be defined by IHC or lesion character was not consistent with the disease time course.

## Discussion

We describe two successful models of experimental infection in cattle with an H5N1 clade 2.3.4.4b genotype B3.13 strain. Signs of clinical disease from a respiratory route of exposure were variable and may not be recognized under field conditions. Experimentally inoculated cows showed clinical signs for 7–14 days, with changes to the milk similar to those documented in field reports, including change in colour from white to yellow, thickening and presence of flakes or clots^2,14^. From 14 to 24 DPI, after cows appeared to be recovering, viral RNA was still detected in the milk and replicating virus was present in the inoculated mammary gland quarters as detected by IHC on 24 DPI. However, viable virus was not found in the pooled milking machine bucket or individual quarter milk samples after 12 DPI. The detection of virus neutralization antibodies in the inoculated quarters coincided with negative virus isolation. Additional functional antibody studies are needed for use with milk as the sample type to understand when neutralizing antibodies appear and how long they last in larger numbers of animals. All calves and cows seroconverted during the study, confirming infection from both routes of inoculation. The commercial ELISA used to detect NP antibodies had not been validated by the manufacturer for bovine serum or milk at the time of this study. The kit cutoff value is 0.5 for avian species and included validation for H5N1 strains. The kit cutoff value is 0.6 for pigs, but based on H1 and H3 subtypes. Robust validation with known positive and known negative bovine samples is needed for establishing a sensitive and specific cutoff for this host and subtype and has now been reported^15^, and owing to the known exposure, consecutive sampling and steady decline of the sample/negative optical density ratios, we used ≤0.6 as the cutoff for positivity.

The amount and duration of virus shed in milk from the inoculated mammary quarters are major findings of this study and point to the mammary gland and milk as primary sources of virus spread within and between dairy herds. This is consistent with the finding that movement of lactating cows was a primary epidemiologic link between the earliest herds involved in the outbreak^4^. Virus replication in infected mammary glands was considerably more than that expected from lungs of animals infected with host-adapted IAV^16^. This is probably, at least in part, due to the cellular structure and physiology of the lung compared to those of the mammary gland. Although IAV-susceptible epithelial cells line the conducting airways, these airways make up a minority of the lung structure. On the basis of both NP IHC from naturally infected dairy cattle and lectin staining, most of the mammary gland is composed of IAV-susceptible epithelial cells^2,17^. Moreover, there is regular sloughing of epithelial cells and secretion of milk fat globules. Following release from the secretory epithelial cell, milk fat globules are bordered by epithelial cell membrane. Milk fat globules can also have a membrane crescent that consists of epithelial cell cytoplasm. Both the epithelial cell membrane and the crescent of milk fat globules may be lined by or contain non-infectious and/or infectious viral particles; however, further exploration of mammary tissue by electron microscopy is needed to confirm the role that milk fat globules play in the shedding of HPAI. The fibrosis and loss of secretory alveoli in the evaluated sections of the left rear mammary gland varied from mild to severe and were more extensive in sections evaluated in cow 2,112. A loss of secretory alveoli was also noted in directly IAV-inoculated mammary glands of nursing ferrets and mothers of IAV-infected infant ferrets^18^. The extent to which secretory alveoli were lost and replaced by fibrous connective tissue within the affected quarter or quarters may account for the variable return to milk production of individual animals following HPAI infection reported by farms^2^. The long-term impact of mammary fibrosis in recovered cows in subsequent lactation cycles remains to be determined. Fibrosis is not a common finding in the lung following uncomplicated IAV infection and supports a divergent immunopathogenesis^18,19^. This may be due to the need for limiting inflammatory responses in an organ that is vital to life, the number of cells susceptible to infection, and mechanisms of host viral clearance through immune responses. This hypothesis is further supported by the systemic clinical signs observed in the dairy cattle that included depression, anorexia and drop in rumen motility. The presence of replicating virus in the inoculated mammary quarters at the time of necropsy at 24 DPI suggests a longer course for this organ to effectively clear the virus in comparison to that of the organs of the respiratory tract in which virus becomes undetectable around 7 DPI (ref. ^19^). Although fibrosis would probably result in decreased milk production for animals in the field, prevention of fibrosis could be a primary variable for vaccine efficacy trials, along with significant reduction in virus titres. The application of a trichrome strain effectively demonstrated the amount of fibrous connective tissue present within glands, and sections could be taken after the duration and quantity of viral shedding were characterized.

No to mild respiratory signs have been reported in many affected dairy herds in the USA^14^ and relatively few bovine respiratory diagnostic samples have been confirmed to be positive for H5N1 HPAI. The clinical signs, RT–qPCR results, macroscopic lesions, histologic findings and antigen distribution of this study align with these field observations. However, the mild macroscopic and histologic lung lesions, detection of replicating IAV by IHC in the lower respiratory tract, and RT–qPCR detection with virus isolation in two upper respiratory swabs confirmed a respiratory phase of infection following aerosol inoculation, similar to a previous study that experimentally inoculated calves with a different genotype of H5N1 (ref. ^20^). Although respiratory infection was limited in the four heifers, the detection of viable virus in two out of four implies that there is a role for the respiratory route as a mode of infection and transmission that may be important in an animal facility that commonly holds hundreds of animals. If natural exposure to respiratory secretions or infected milk leads to production of neutralizing antibodies, it may prove beneficial in preventing mastitis if the cow is subsequently re-exposed by the intramammary route during the milking process.

The enteric signs that included both diarrhoea (cow 2,129) and dry faecal material (cow 2,112) noted in this study also align with reports from the field and justify the initial clinical differential diagnosis of an atypical enteric bovine coronavirus infection for the milk drop syndrome. Minimal lesions were observed in the jejunum of both cows and cannot be definitively attributed to HPAI H5N1 infection. The loss of crypts and expansion of the lamina propria by fibrous connective tissue is a chronic change present at 24 DPI, and owing to study timing, tissues were collected days after enteric signs were noted. RT–qPCR did not detect HPAI in any rectal swab at any time point or in the jejunum or faeces of inoculated animals at necropsy, but low levels of replication in the upper gastrointestinal system cannot be ruled out on the basis of the timing of the necropsy. Additional studies focused on pathologic assessment and antigen distribution at multiple time points following inoculation should be conducted to more thoroughly evaluate tissue tropism at the cellular level.

The interspecies transmission of H5N1 clade 2.3.4.4b to many mammal species, now including cattle with genotype B3.13, is unprecedented in our understanding of avian-adapted IAV^21,22^. This raises concern for other mammalian hosts, including pigs and other livestock and pets, and particularly for humans. The human cases in the USA have been clinically mild and limited in number^6,7^, but concern remains as H5N1 continues to expand into new hosts, spread geographically and reassort with other avian or mammalian subtypes. The possibility of H5N1 becoming endemic in cattle increases as the number of infected herds continues to rise^1^. Pasteurization was shown to inactivate virus, and retail milk remains negative for infectious virus, and thus is not a risk for human consumption when processed according to Food and Drug Administration standards^23,24^. Unpasteurized milk and dairy products are a risk to humans and other animals. Milk from H5N1-positive dairy herds or from suspect cows that is diverted from the human food supply should not be fed to other farm animals and it should not be dumped without inactivation owing to the risk to surrounding farms or peridomestic wild animals.

The sustained transmission among dairy cattle is an animal health crisis owing to production and economic losses and is a public health challenge owing to occupational exposure on dairy farms and human pandemic potential. The development of reproducible experimental challenge models such as the ones described here is the essential first step to inform subsequent research on intervention and vaccination strategies. Although our study was limited in the number of animals owing to their size and high containment space requirements, we reproduced the clinical observations from the field of viral mastitis due to HPAI H5N1 infection alone and confirmed respiratory involvement. Further studies to understand transmission, refine the pathogenesis model and define the kinetics of protective immunity in cattle infected with HPAI are urgently needed.

## Methods

### Strain characterization

We analysed the HPAI H5N1 genotype B3.13 virus used this study (A/dairy cattle/Texas/24-008749-002/2024: TX/24: National Center for Biotechnology Information (NCBI) PP755581–PP755588) with other B3.13 strains generated in ref. ^4^ and newly sequenced data collected between 16 April and 8 May 2024. IAV RNA from samples was amplified^25^; then we generated cDNA libraries by using the Illumina DNA Sample Preparation Kit, (M) Tagmentation (Illumina) and either the iSeq or NextSeq Reagent Kit v2 (Illumina) according to the manufacturer’s instructions. We performed reference-guided assembly of genome sequences using IRMA v1.1.5 (ref. ^26^). We aligned each gene segment using mafft v7.490 (ref. ^27^) and inferred maximum-likelihood phylogenetic trees for each gene segment as well as concatenated whole genomes using IQ-Tree v2.2.2 (ref. ^28^). These phylogenetic gene trees were used to determine how representative the TX/24 strain was relative to viruses collected between March and May 2024. This approach implemented the evaluate algorithm in PARNAS^29^ that objectively identified the most representative strain in the B3.13 group and how near the TX/24 strain was relative to that representative strain.

### Biosafety risk mitigation

Work with the HPAI H5N1 strain and subsequent animal samples was conducted in secure biosafety level 3 (BSL3) laboratory space following the Biosafety in Microbiological and Biomedical Laboratories, 6th edition^30^, guidelines. All animal studies were carried out in a BSL3-Agriculture facility in compliance with the Institutional Animal Care and Use Committee of the National Animal Disease Center (NADC) of the US Department of Agriculture (USDA) and the Agriculture Research Service (ARS), and Federal Select Agent Program regulations.

### Cow intramammary inoculation

**Extended Data Fig. 4:**
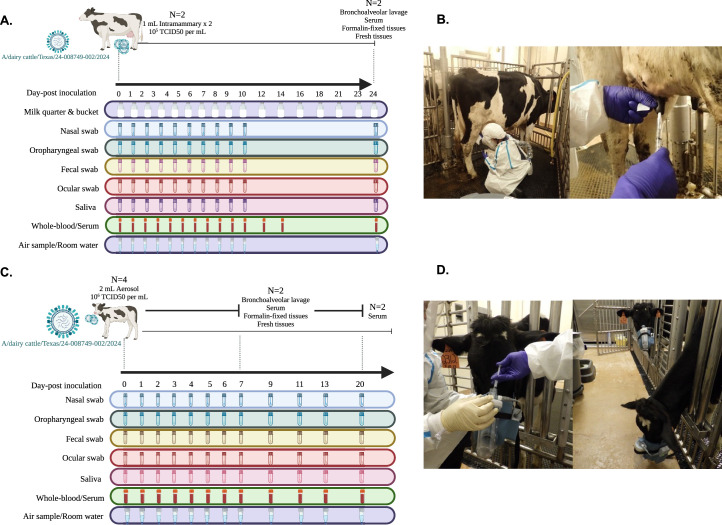
Inoculation of Holstein calves and cows. A) Schematic of lactating cow study design. B) Photographs of intramammary inoculation in the lactating Holstein cows via teat canula. C) Schematic of heifer calf study design. D) Photographs of aerosol respiratory inoculation in the Holstein heifer calves using nebulizer masks. The graphics in **a**,**c** were created with BioRender.com.

The A/dairy cattle/Texas/24-008749-002/2024 virus was isolated from a milk sample from the outbreak investigation in 10-day-old embryonating chicken eggs^25^ and subsequently titrated on the London line of Madin–Darby canine kidney (MDCK, International Reagent Resource) cells. Two healthy non-pregnant, lactating Holstein cows of approximately 3 years of age and 280 days in milk during their first lactation were moved from the on-site isolated NADC dairy into BSL3-Agriculture containment and acclimated for approximately 1 week before inoculation. Cows were inoculated with 1 ml of 1 × 10^5^ tissue culture infectious dose 50 (TCID_50_) per millilitre of the first passage of egg-grown A/dairy cattle/Texas/24-008749-002/2024 by an intramammary route in each of two quarters, front right and rear left, using a teat canula (Extended Data Fig. 4a,b). The inoculation occurred after milking and standard teat disinfection, and the teats were additionally disinfected with an alcohol wipe immediately before the procedure. After instilling, the inoculum was moved upwards into the teat sinus with gentle massage.

### Calf aerosol inoculation

Five healthy Holstein heifer calves of approximately 1 year of age born in isolation on the facility were either inoculated with 2 ml of 1 × 10^6^ TCID_50_ per millilitre of A/dairy cattle/Texas/24-008749-002/2024 (*n* = 4) from the same virus stock as the cow inoculum or sham-inoculated with phosphate-buffered saline (PBS; *n* = 1) by an aerosol respiratory route. For aerosol inoculation, calves were restrained in a cattle stanchion and the inoculum was delivered by nebulization into a mask covering the nostrils and mouth (Extended Data Fig. 4c,d). A 2 ml volume of inoculum was placed into the medicine cup of the portable equine nebulizer (Flexineb E3, Nortev). Nebulization continued until all of the inoculum was delivered (approximately 3 min). Following the inoculum, 2 ml sterile PBS was nebulized through the apparatus. The portable equine nebulizer uses a vibrating mesh nebulizer, which generates aerosol droplets in the respirable range. Droplets generated by vibrating mesh nebulizers were shown to be 3–5 μm (ref. ^31^), with the manufacturer of the nebulizer reporting that 70% of the total nebulized volume resulted in droplets of <5 μm. Aerosolized droplets that are less than 5 μm bypass the upper respiratory tract and are deposited deep in the lower respiratory tract^32^.

### Clinical evaluation and sample collection

In the lactating cows, rumination and other behaviours were monitored continuously using an ear-tag accelerometer sensor (CowManager SensOor, Agis Automatisering). For cows and heifers, clinical signs were visually monitored and recorded daily (Extended Data Table 5), including body temperature with a thermal microchip, respiratory effort and rate, nasal and ocular discharge, and faecal consistency. The lactating cows were milked once daily in the morning using a portable milker (Hamby). Before milking, the teats were cleaned with water, disposable paper towels, and isopropyl alcohol wipes, and milk was manually stripped from each teat into separate 50-ml tubes. The milk samples were evaluated by quarter for mastitis by the California mastitis test (ImmuCell) and for consistency and colour using a dental colour scorecard on a scale of 0–12. The remaining milk stripped by quarter was used for virus and/or antibody detection. After milking, a sample from each cow’s milking machine bucket was also collected. A separate milking claw was used for each cow, and the claw, tubing and bucket were washed, degreased and disinfected (Virkon S, LanXess) after each use.

The remaining clinical samples were collected daily from cows and calves for 7 DPI and approximately every 2 days thereafter until study termination. To sample the deep pharyngeal region, a Frick cattle mouth speculum was used. Using manual restraint, the speculum was placed over the tongue to the pharynx and held there. Through the speculum, FLOQ nylon-tipped (Copan) or long cotton-tipped (SCA Health) swabs were used to collect samples of the deep oropharynx. Ocular, nasal and rectal samples were collected with FLOQ nylon swabs. Whole blood was collected through jugular venipuncture and placed into molecular transport medium (PrimeStore MTM, Longhorn) and into serum separator tubes. Saliva was collected with an absorbent pad (Super-SAL, Oasis Diagnostics).

### Pathologic evaluation

All cattle were euthanized through intravenous administration of pentobarbital sodium (Fatal Plus, Vortech Pharmaceuticals). Heifers 2,311 and 2,316 and a negative control heifer were necropsied at 7 DPI, whereas the remaining two aerosol-inoculated heifers were necropsied at 20 DPI. The lactating dairy cows were necropsied at 24 DPI. At the time of necropsy, the thoracic cavity, abdominal cavity and cranium (longitudinal section) underwent macroscopic evaluation. Paired fresh and formalin-fixed tissues for RT–qPCR and microscopic evaluation, respectively, were collected (Extended Data Table 1). Formalin-fixed tissues were processed routinely for microscopic evaluation. Additional fresh samples included aqueous and vitreous humour, urine, faeces and rumen contents.

IHC targeting the NP antigen (1:2,000, GeneTex, GTX125989) of IAV was performed as previously described with the inclusion of positive (lung from an IAV-inoculated pig) and negative tissue (respiratory tissues from the control heifer and lung from a non-IAV-inoculated pig) controls^16^. Nasal turbinate, trachea, lung, tracheobronchial lymph node, supramammary lymph node (lactating cows) and individual mammary quarters (lactating cows) from each animal were selected for IHC. Additional tissue sections with RT–qPCR detection and/or histologic lesions were also evaluated by IHC for the detection of NP antigen to confirm a causal link between IAV and the lesion. A Masson’s trichrome stain (Newcomer Supply) was performed as per the manufacturer’s instructions on representative mammary gland sections from each of the four quarters to highlight fibrous connective tissue. Sections were examined by a veterinary pathologist using an Olympus BX43 light microscope. Photomicrographs were taken using an Olympus DP28 camera and Olympus cellSens Standard.

### Virus detection in clinical samples

IAV RNA extraction and RT–qPCR were performed at the USDA National Veterinary Services Laboratories (NVSL) according to the standard operating procedures as previously described^5,33^. Clinical ante-mortem and necropsy samples were tested using an IAV matrix gene RT–qPCR. A subset of positive samples with Ct ≤ 35 was processed for whole-genome sequencing with previously described methods^5^; and after amplification was completed, we generated cDNA libraries for MiSeq as indicated under virus characterization. A selection of RT–qPCR positive samples with Ct < 30 or milk samples targeted to address impact of immunity on virus isolation were inoculated onto 10-day-old embryonating chicken eggs to determine the presence of viable virus.

### Antibody detection

Seroconversion was determined using a blocking ELISA to detect antibodies to the NP (influenza A antibody, IDEXX) according to the manufacturer’s instructions for swine at the time of the study with a 1:10 starting dilution of serum or rennet-treated milk and a cutoff sample/negative optical density ratio of 0.6. Before ELISA, milk samples were treated with rennet (*Mucor miehei*, Sigma-Aldrich) as previously described^34^. H5-specific HI with rooster red blood cells and homologous virus and virus neutralization antibody assays on the London line of MDCK cells with a first passage of MDCK-propagated A/dairy cattle/Texas/24-008749-002/2024 were conducted as previously described^35–37^. Before HI, sera were heat-inactivated at 56 °C for 30 min, treated with receptor-destroying enzyme (Hardy Diagnostics) and adsorbed with 0.5% and subsequently 100% rooster red blood cells for 20 min each to remove nonspecific haemagglutinin inhibitors and natural serum agglutinins.

### Processing of raw sequence data and single nucleotide variant calling

To analyse the Illumina short-read data for 82 samples, we used the Flumina pipeline (https://github.com/flu-crew/Flumina) for processing and analysing influenza data^4^. The pipeline uses Python v3.10, R v4.4 (R Development Core Team 2024), and SnakeMake^38^ to organize programs and script execution. The reads are preprocessed using FASTP^39^, removing adaptor contamination, low-complexity sequences and other artefacts. Consensus contigs for phylogenetics were assembled using IRMA v1.1.4 (ref. ^26^). The pipeline maps cleaned reads to the reference cattle strain derived from sequencing the stock inoculum of A/dairy cattle/Texas/24-008749-002/2024 using BWA (bwa index –a bwtsw)^40^. High-frequency single nucleotide variants (SNVs) were called with GATK v.4.4 (ref. ^41^), and low-frequency SNVs were called with LoFreq^42^. To assess potential SNV phenotypic changes, a database was generated using the Sequence Feature Variant Types tool from the Influenza Research Database^43^ for all eight genes. To estimate genome-wide natural selection, we used the program SNPGenie on the VCF files^44^.

### Statistical analysis

Pearson correlation coefficients between Ct values for milk samples from the bucket as well as the inoculated teats (front right and rear left) and rumination time, milk production, and consistency and colour scores of milk from the inoculated teats for all time points were calculating using the package Hmisc v. 5.1-3c in RStudio 2022.02.1+461 ‘Prairie Trillium’. Correlations were considered significant at *P* < 0.05 and visualized using the package corrplot v0.92.

### Reporting summary

Further information on research design is available in the Nature Portfolio Reporting Summary linked to this article.

## Online content

Any methods, additional references, Nature Portfolio reporting summaries, source data, extended data, supplementary information, acknowledgements, peer review information; details of author contributions and competing interests; and statements of data and code availability are available at 10.1038/s41586-024-08166-6.

## Supplementary information

Reporting Summary

## Figures and Tables

**Extended Data Table 1:**
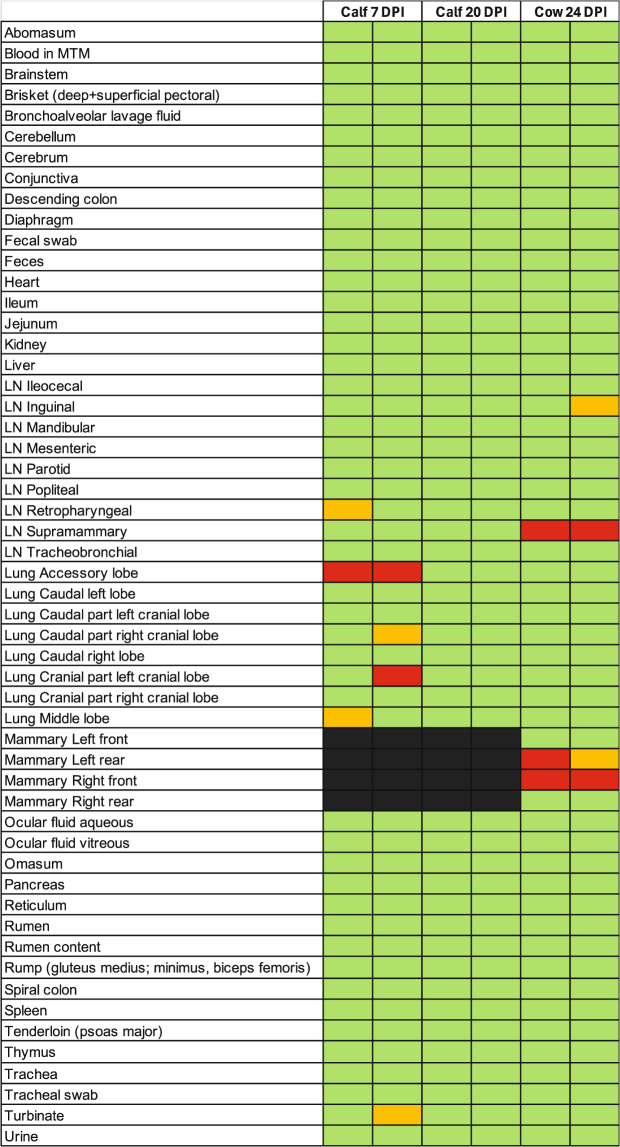
Tissues and samples collected at necropsy and tested by RT-qPCR*

*Each column represents an individual animal. Green = Ct>38, orange = Ct 35-38, red = Ct <35, black = not collected.

**Extended Data Table 2:**
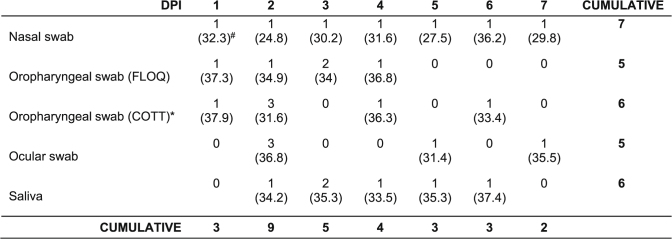
Positive RT-qPCR samples in aerosol inoculated heifer calves*

#Number of samples with Ct < 38 out of four and RT-qPCR Ct value or average in parentheses.

*Virus isolation was unsuccessful for attempted cotton oropharyngeal swabs. Five FLOQ nasal swabs from 2316 and one FLOQ oropharyngeal swab from 2311 were positive by virus isolation.

**Extended Data Table 3:**
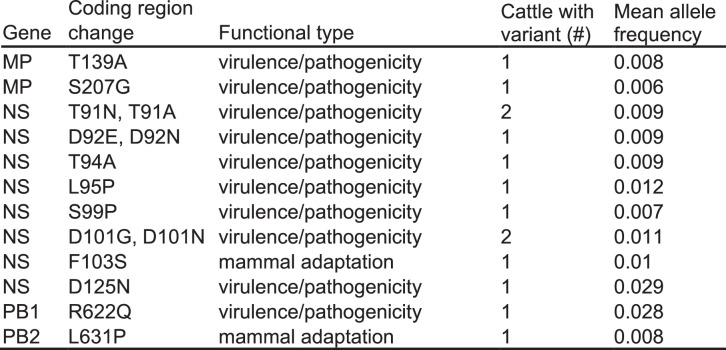
Raw read data collected from cattle were processed and high- and low-frequency single nucleotide variants (SNVs) were identified

**Extended Data Table 4:**
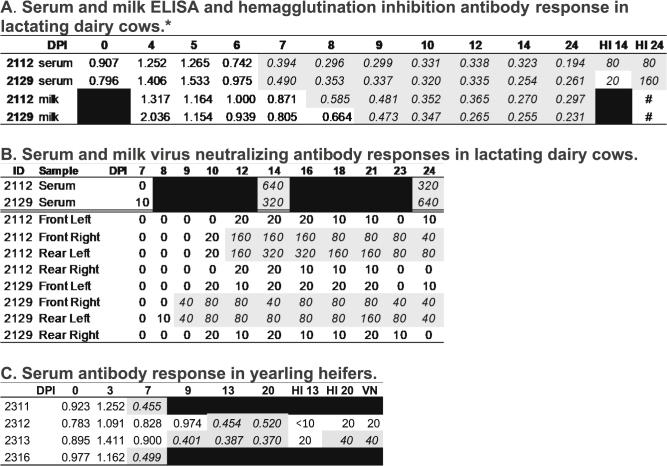
Antibodies detected in cows and heifers

A. Serum and milk ELISA and hemagglutination inhibition antibody response in lactating dairy cows.*

*NP ELISA sample to negative (S/N) ratio of optical densities reported for days post inoculation (DPI) indicated in header row. Hemagglutination inhibition (HI) assay reported as reciprocal titer on 14 and 24 DPI. Positive samples indicated by gray shading and italics; black indicates not tested.

#ELISA S/N reported for pooled milk bucket samples. HI titers in milk at 24 DPI varied by quarter between 10-40 (see data file).

**B. Serum and milk virus neutralizing antibody responses in lactating dairy cows**.

*Virus neutralization assays reported as reciprocal titers for days post inoculation (DPI) indicated in header row for serum and milk from each mammary quarter. Positive samples indicated by gray shading and italics; black indicates not tested.

**C. Serum antibody response in yearling heifers**.

*NP ELISA sample to negative (S/N) ratio of optical densities reported for days post inoculation (DPI) indicated in header row. Hemagglutination inhibition (HI) on 13 and 20 DPI and virus neutralization (VN) on 20 DPI reported as reciprocal titer. Positive samples indicated by gray shading and italics; black indicates not tested. Two animals were euthanized on 7 DPI.

**Extended Data Table 5:**
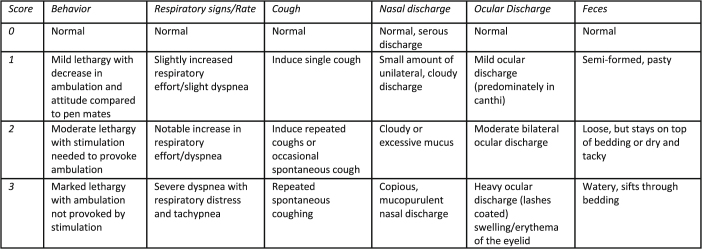
Clinical scores used to assess dairy calves and lactating dairy cows*

*Adapted from https://www.vetmed.wisc.edu/fapm/svm-dairy-apps/calf-health-scorer-chs/ Calf Health Scorer (CHS) – Food Animal Production Medicine – UW–Madison (wisc.edu).

## Data Availability

Data that support the findings of the clinical challenge studies (clinical-challenge-study-data file), sequence data and materials used in the analysis are available via Zenodo at https://zenodo.org/doi/10.5281/zenodo.13126628 (ref. ^45^). NCBI GenBank accession numbers for sequence data used in the analyses and the raw read sequence data from experimental samples are accessible via Zenodo at https://zenodo.org/doi/10.5281/zenodo.13126628 (ref. ^45^). Raw sequence data are available in the NCBI Sequence Read Archive, under Bioproject accession number PRJNA1102327.

## Code availability

Code used in the analysis is available via Zenodo at https://zenodo.org/doi/10.5281/zenodo.13126628 (ref. ^45^).
