# Supplementary material for: Dairy cows inoculated with highly pathogenic avian influenza virus H5N1
Source: Nature. 2024 Oct 15;637(8047):913–20. doi: 10.1038/s41586-024-08166-6 (PMC11754099; doi:10.1038/s41586-024-08166-6)
Supplement: Supplementary file 1 — Reporting Summary [file 41586_2024_8166_MOESM1_ESM.pdf]

nature portfolio

Corresponding author(s): Amy L. Baker

Last updated by author(s): Sep 13, 2024

Reporting Summary

Nature Portfolio wishes to improve the reproducibility of the work that we publish. This form provides structure for consistency and transparency in reporting. For further information on Nature Portfolio policies, see our Editorial Policies and the Editorial Policy Checklist.

Statistics

For all statistical analyses, confirm that the following items are present in the figure legend, table legend, main text, or Methods section.

n/a	Confirmed	
☐	☒	The exact sample size (n) for each experimental group/condition, given as a discrete number and unit of measurement
☒	☐	A statement on whether measurements were taken from distinct samples or whether the same sample was measured repeatedly
☐	☒	The statistical test(s) used AND whether they are one- or two-sided<br>*Only common tests should be described solely by name; describe more complex techniques in the Methods section.*
☐	☒	A description of all covariates tested
☐	☒	A description of any assumptions or corrections, such as tests of normality and adjustment for multiple comparisons
☐	☒	A full description of the statistical parameters including central tendency (e.g. means) or other basic estimates (e.g. regression coefficient) AND variation (e.g. standard deviation) or associated estimates of uncertainty (e.g. confidence intervals)
☐	☒	For null hypothesis testing, the test statistic (e.g. F, t, r) with confidence intervals, effect sizes, degrees of freedom and P value noted<br>*Give P values as exact values whenever suitable.*
☒	☐	For Bayesian analysis, information on the choice of priors and Markov chain Monte Carlo settings
☒	☐	For hierarchical and complex designs, identification of the appropriate level for tests and full reporting of outcomes
☐	☒	Estimates of effect sizes (e.g. Cohen's d, Pearson's r), indicating how they were calculated

Our web collection on statistics for biologists contains articles on many of the points above.

Software and code

Policy information about availability of computer code

Data collection	None
Data analysis	RStudio 2022.02.1+461 "Prairie Trillium", bwa=0.7.17, bioconductor-biostrings=2.66.0, fastp=0.23.4, gatk4=4.5.0.0, lofreq=2.1.5, python=3.10.14, samtools=1.19, r-doparallel=1.0.17, r-ape=5.7_1, r-data.table=1.14.8, r-essentials=4.2, r-seqinr=4.2_30, r-stringr=1.5.0, r-foreach=1.5.2, snakemake=7.32.4, IRMA v1.1.5, mafft v7.490, IQ-Tree v2.2.2, PARNAS v0.1.5

For manuscripts utilizing custom algorithms or software that are central to the research but not yet described in published literature, software must be made available to editors and reviewers. We strongly encourage code deposition in a community repository (e.g. GitHub). See the Nature Portfolio guidelines for submitting code & software for further information.

Data

Policy information about availability of data

All manuscripts must include a data availability statement. This statement should provide the following information, where applicable:

- Accession codes, unique identifiers, or web links for publicly available datasets
- A description of any restrictions on data availability
- For clinical datasets or third party data, please ensure that the statement adheres to our policy

Data that support the findings of the clinical challenge studies and sequence data, code, and materials used in the analysis are available at https://github.com/flu-crew/dairy-cattle-hpai-experiment DOI:10.5281/zenodo.13126629.

Research involving human participants, their data, or biological material

Policy information about studies with human participants or human data. See also policy information about sex, gender (identity/presentation), and sexual orientation and race, ethnicity and racism.

Reporting on sex and gender	N/A
Reporting on race, ethnicity, or other socially relevant groupings	N/A
Population characteristics	N/A
Recruitment	N/A
Ethics oversight	N/A

Note that full information on the approval of the study protocol must also be provided in the manuscript.

Field-specific reporting

Please select the one below that is the best fit for your research. If you are not sure, read the appropriate sections before making your selection.

☒ Life sciences ☐ Behavioural & social sciences ☐ Ecological, evolutionary & environmental sciences

For a reference copy of the document with all sections, see nature.com/documents/nr-reporting-summary-flat.pdf

Life sciences study design

All studies must disclose on these points even when the disclosure is negative.

Sample size	5 yearling heifers and 2 lactating Holstein dairy cows. No sample size calculation was performed. The groups sizes were determined by space requirements in the BSL3-Ag high containment facility.
Data exclusions	None.
Replication	Due to the restriction of BSL3-Ag containment, the study sample size is limited and there were no replicates.
Randomization	Animals were randomly chosen based upon availability.
Blinding	Blinding was not possible but not necessary as tests were not subjective.

Reporting for specific materials, systems and methods

We require information from authors about some types of materials, experimental systems and methods used in many studies. Here, indicate whether each material, system or method listed is relevant to your study. If you are not sure if a list item applies to your research, read the appropriate section before selecting a response.

Materials & experimental systems

n/a	Involved in the study	
☐	☒	Antibodies
☐	☒	Eukaryotic cell lines
☒	☐	Palaeontology and archaeology
☐	☒	Animals and other organisms
☒	☐	Clinical data
☒	☐	Dual use research of concern
☒	☐	Plants

Methods

n/a	Involved in the study	
☒	☐	ChIP-seq
☒	☐	Flow cytometry
☒	☐	MRI-based neuroimaging

Antibodies

Antibodies used	rabbit polyclonal anti-influenza A NP (GeneTex, https://www.genetex.com) used for IHC at 1:2000 dilution
Validation	https://www.genetex.com/MarketingMaterial/Index/five_pillars

Eukaryotic cell lines

Policy information about cell lines and Sex and Gender in Research

Cell line source(s)	Madin-Darby Canine Kidney cells, London Line (Cat # FR-58, International Reagent Resource)
Authentication	Cells were not authenticated
Mycoplasma contamination	Cells were not tested for mycoplasma
Commonly misidentified lines (See ICLAC register)	None

Animals and other research organisms

Policy information about studies involving animals; ARRIVE guidelines recommended for reporting animal research, and Sex and Gender in Research

Laboratory animals	Holstein cattle of 1 year of age (n=5) and 3 years of age (n=2)
Wild animals	None
Reporting on sex	All female due to nature of disease
Field-collected samples	None
Ethics oversight	Approved by the institutional animal care and use committee of the USDA ARS National Animal Disease Center

Note that full information on the approval of the study protocol must also be provided in the manuscript.

Plants

Seed stocks	N/A
Novel plant genotypes	N/A
Authentication	N/A
